# Genome Sequencing of a *Fusarium* Endophytic Isolate from Hazelnut: Phylogenetic and Metabolomic Implications

**DOI:** 10.3390/ijms26094377

**Published:** 2025-05-05

**Authors:** Andrea Becchimanzi, Beata Zimowska, Marina Maura Calandrelli, Luigi De Masi, Rosario Nicoletti

**Affiliations:** 1 Department of Agricultural Sciences, University of Naples Federico II, 80055 Portici, Italy; andrea.becchimanzi@unina.it; 2 Department of Plant Protection, University of Life Sciences, 20-069 Lublin, Poland; beata.zimowska@up.lublin.pl; 3 Research Institute on Terrestrial Ecosystems (IRET), National Research Council (CNR), 80131 Napoli, Italy; marinamaura.calandrelli@cnr.it; 4 Institute of Biosciences and Bioresources (IBBR), National Research Council (CNR), 80055 Portici, Italy; 5 Research Centre for Olive, Fruit and Citrus Crops, Council for Agricultural Research and Economics, 81100 Caserta, Italy; rosario.nicoletti@crea.gov.it

**Keywords:** biosynthetic gene clusters, defensive mutualism, endophytes, *Fusarium citricola* species complex, mycotoxins, phylogenesis

## Abstract

This study reports on the whole genome sequencing of the hazelnut endophytic *Fusarium* isolate Hzn5 from Poland. It was identified as a member of the *Fusarium citricola* species complex based on a phylogenetic analysis which also pointed out that other hazelnut isolates, previously identified as *F. lateritium* and *F. tricinctum*, actually belong to this species complex. Genome annotation allowed the mapping of 4491 different protein sequences to the genome assembly. A further in silico search for their potential biosynthetic activity showed that predicted genes are involved in 1110 metabolic pathways. Moreover, the analysis of the genome sequence carried out in comparison to another isolate, previously identified as an agent of hazelnut gray necrosis in Italy, revealed a homology to several regions containing biosynthetic gene clusters for bioactive secondary metabolites. The resulting indications for the biosynthetic aptitude concerning some emerging mycotoxins, such as the enniatins and culmorin, should be taken into consideration with reference to the possible contamination of hazelnuts and derived products.

## 1. Introduction

The filamentous fungi of the genus *Fusarium* (Sordariomycetes, Hypocreales, Nectriaceae) are widespread in every environment on Earth and are frequently isolated from all sorts of host organisms with which they may establish a variety of antagonistic or mutualistic interactions. This pervasive ecological occurrence reflects a huge taxonomic variation, which is attested by the ongoing classification of novel species [1]. Indeed, the recent spread of biomolecular tools has enabled researchers in the field to perform accurate identifications and phylogenetic reconstructions. While contributing to the resolution of the intricate taxonomy of these fungi, recent findings also emphasize the importance of a correct classification in applicative terms, to implement their containment and control protocols [2].

Hazelnut (*Corylus avellana* L.) represents a meaningful example of how misidentifications and taxonomic adjustments may interfere in the assessment of the real ecological role by components of the mycobiome. Even if not generally considered among the key pathogens of hazelnut, *Fusarium* spp. have a widespread occurrence in association with this tree crop and may impact plant health and product quality [3,4,5]. In fact, these fungi were identified as agents of the nut gray necrosis (NGN) [6], negatively affecting production both in quantitative terms and as a result of mycotoxin contamination of kernels [7]. On the other hand, the endophytic habit may entail positive effects on plant health through the implementation of defensive mutualism. This symbiotic relationship is particularly considered in view of possible exploitation for reducing the impact of chemicals in crop management, above all in the case of semi-extensive crops such as hazelnut [8]. Indeed, one of the basic aspects characterizing defensive mutualism consists in the release of antimicrobial products in the host tissues; undoubtedly, *Fusarium* represents one of the best-investigated fungal genera in this respect [9].

In view of developing the available information on the classification and the ecological role of hazelnut-associated *Fusaria*, in this research we further characterize an endophytic isolate (Hzn5) from Poland. Given the importance of the topic, particular interest has been paid to both the phylogenetic relationships with the known species in the *Fusarium citricola* species complex (FCCSC) and the secondary metabolite (SM) biosynthetic potential. The high-quality genome sequencing of Hzn5 is reported, along with its de novo assembly and the annotation of the biosynthetic gene clusters (BGCs) encoding proteins involved in the biochemical pathways related to the main *Fusarium* SMs. This work was carried out in comparison with an Italian isolate (PT) reported as an NGN agent and ascribed to the species *F. tricinctum* [10], representing the only *Fusarium* strain from hazelnut for which a draft genome sequence is publicly available.

## 2. Results

### 2.1. Morphological Description

Morphological characters of isolate Hzn5 were previously studied [3] and now further assessed in comparison to the known species in the FCCSC [11]. Some peculiar traits were observed in Hzn5 consisting in the production of abundant chlamydospores and the lack of microconidia. Within the FCCSC, only *F. celtidicola* is reported to form chlamydospores, but this species also produces microconidia. Furthermore, our strain seems to be unique within the FCCSC by lacking red pigments and forming sclerotia (Figure S1). A more detailed description is provided below, which could be relevant for further taxonomic assessments following the recent evidence that many strains deposited as *F. lateritium* in several collections actually belong to the FCCSC [12].

Isolate Hzn5, *Fusarium* sp.: colonies at 25 ± 1 °C reaching 45–47 mm after 10 days on PDA, 78–85 mm on SNA; aerial mycelium on PDA abundant and dense, floccose to wooly with crenate margin, white-cream colored; reverse, salmon-pink, darkening at the center, without medium pigmentation; dark gray sclerotia-like structures visible in 30-day cultures on PDA; macroconidia abundantly formed in orange sporodochia from monophialidic conidiogenous cells, 3–5-septate, predominantly 3-septate, unequally curved; apex pointed, base poorly developed, foot-shaped, hyaline; size: 5-septate: (27–)28–29.5 x (3–)3.5–5.5 μm; 4-septate: (25–)26–27.5 x (3–)3.5–5 μm; 3-septate: (22.5–)24–25.5 x (2.5–)3–4.5 μm; microconidia absent; chlamydospores abundant, formed quickly, mainly in chains, but also single or paired, smooth-walled, intercalary, globose or subglobose to pyriform.

### 2.2. Phylogenesis

**Figure 1:**
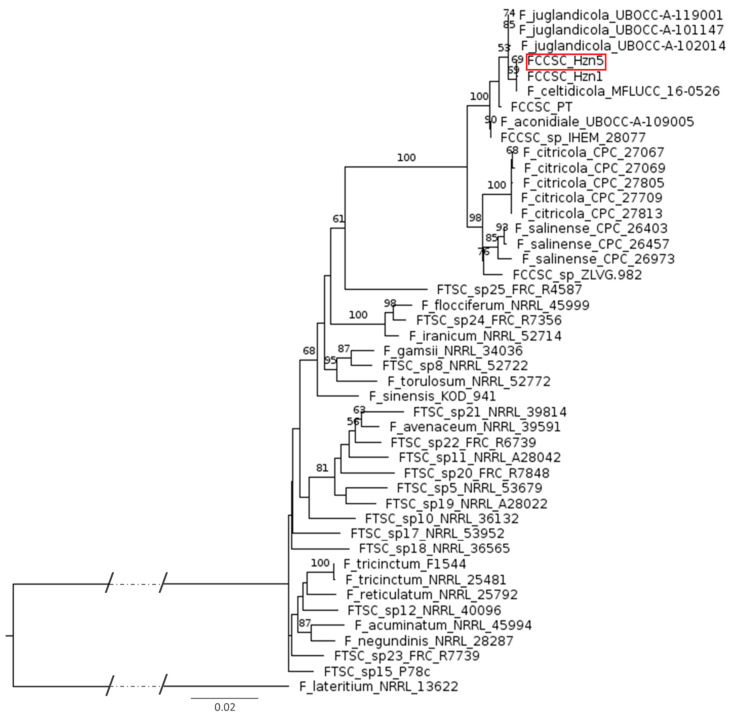
Phylogram representing the relationships among the hazelnut isolates, species in the FCCSC and the FTSC, and a reference strain of *F. lateritium*. The tree was based on maximum likelihood (ML) analysis of combined *tef1* and *rpb2* sequences. Bootstrap support values ≥ 50% for ML are presented above branches. The scale bar indicates the number of nucleotide substitutions per site; the dotted lines represent 0.08 nucleotide substitution per site.

The above distinctive morphological features could assume the finding of a new *Fusarium* species. Therefore, a phylogenetic analysis was carried out based on the relevant molecular markers considered for identifying species in the FCCSC, namely the translation elongation factor 1-alpha (*tef1*) and the DNA-directed RNA polymerase II core subunit (*rpb2*) [10]. The generated phylogram included several FCCSC representatives, all the taxa recognized within the *F. tricinctum* species complex (FTSC) in the recent revision by Laraba et al. [13], and a reference strain of *F. lateritium* (Table S1). As it is shown in Figure 1, the hazelnut endophytic isolates are closely related to *F. celtidicola* and are part of a clade also including strains of *F. juglandicola* and *F. aconidiale*, as well as the PT isolate from symptomatic hazelnut. Besides showing an evident distance from *F. lateritium*, this phylogram clearly depicts the close relatedness between the FCCSC and the FTSC. Within this proximity, the taxonomic position of FTSC sp. 25, represented by a single strain, should be better considered, as it is placed at the same phylogenetic distance as the whole FCCSC from the other species forming the FTSC.

### 2.3. Genome De Novo Sequence Assembly

Genome de novo analysis of isolate Hzn5 included a quality check of the 59,076,306 paired-end reads obtained from the raw sequencing data, where each base was with a Phred quality score (q or Q) > 30. FastQC tool showed sequence quality modules per base about raw sequencing data both forward (R1) and reverse (R2) from the fastq file format (Figure S2) used for storing the output of high-throughput sequencing instruments, in which sequence letter and quality score are each encoded for brevity with a single character. The sequencing data were deposited in the GenBank database at NCBI and identified with SRA accession number SRR31988993, BioSample SAMN46231596, within the BioProject PRJNA1210087. The best k-mer length estimated was 121 with the highest scaffold N50. The genome de novo assembly was performed by ABySS 2.0 obtaining 10,271 contigs (187 with contig size > 500 bp) and 10,185 scaffolds (140 with scaffold size > 500 bp). Genome assembly quality and completeness were evaluated by QUAST and BUSCO tools, respectively [14,15]. QUAST showed that the maximum contig length was 1,776,231 nt, whereas the maximum scaffold length was 5,912,375 nt with N50 of 1,148,930 and 2,818,754 for contigs and scaffolds, respectively (Figure S3A,B). The cumulative length of contigs and scaffolds was 41,543,006 nt and 41,544,408 nt, respectively. BUSCO’s results on scaffolds are simplified into categories of complete and single-copy (S = 99.2%), complete and duplicated (D = 0.1%), fragmented (F = 0.1%), and missing (M = 0.6%) BUSCO marker genes (BUSCOs) on a total of 4494 BUSCO groups searched, of which 187 contain internal stop codons. The results in BUSCO notation are as follows: C:99.3% (S:99.2%, D:0.1%), F:0.1%, M:0.6%, n: 4494 on the dataset of single-copy orthologs at the Hypocreales order level. BUSCO evaluated our genome assembly of high quality, containing almost all the expected single-copy genes (99.2%) with a very low duplication level (0.1%). BLASTn allowed the annotation of the scaffolds [16] producing 38,307 positive matches in the Hzn5 genome (Table S2). At the same time, a genome annotation using BUSCO’s algorithm Miniprot allowed the mapping of 4491 different protein sequences to the genome assembly (Table S3). Finally, a functional analysis was performed using the OmicsBox software ver. 3.2 (BioBam Bioinformatics, Valencia, Spain), starting from the BLASTn alignments previously obtained. In particular, the InterPro tool searched for protein families (Figure S4A,B), domains (Figure S5), and key amino acidic sites (Figure S6). An analysis in the KEGG, plant reactome, and reactome databases showed that predicted genes are involved in 1110 metabolic pathways (Table S4): 110 KEGG (Figure S7), 649 plant reactome (Figure S8), and 351 reactome pathways (Figure S9).

### 2.4. Annotation of Biosynthetic Gene Clusters Related to Secondary Metabolite Production

As analyzed through the Antibiotics and Secondary Metabolites Analysis Shell database (antiSMASH), a widely used tool in genome mining for SMs [17], 49 BGCs encoding for the synthesis of SMs were found in the genome of isolate Hzn5. Most of them corresponded to terpenes, ribosomally synthesized and post-translationally modified peptides (RiPPs), non-ribosomal peptide synthetases (NRPS), and polyketide synthases (T1PKS, T3PKS) of common occurrence in *Fusarium* [18,19]; twenty-two were identified as known BGCs (Table 1). A comparison with the PT genome [10] indicated that most BGCs are shared between the two strains, with a seemingly complete correspondence for those related to orcinol/orsellinic acid, choline, fusaridione A, ilicicolins, gibepyrone A, bassianolide, chrysogine, bikaverin, ACT-toxin II, squalestatin S1, α-acorenol, and koraiol; these BGCs are commonly identified in the genome of *Fusarium* species [20,21,22,23,24,25,26,27]. Coherent with the genetic distance between the two strains and their possible ascription to different species are the lower similarities observed for the other identified BGCs, namely fusarielin, gibberellin, fujikurins, fofonochlorin, and oxyjavanicin, as well as the absence in PT of the BGC encoding fusarubin/1233A−B/NG-391/lucilactaene.

**Figure 2:**
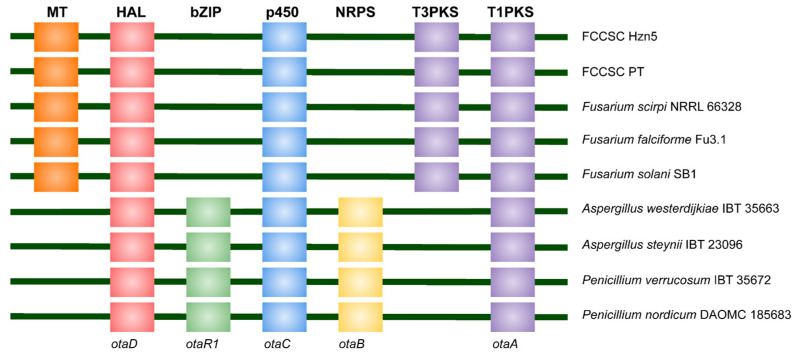
Comparison of the main genes and their related enzymes in the BGCs of 6-hydroxymellein (*Fusarium*) and ochratoxin A (*Aspergillus* and *Penicillium*) producers. Besides Hzn5 and PT, the GenBank reference genomes of each species were examined. Gene and protein abbreviations as used in [29].

The BGC corresponding to 6-hydroxymellein was not previously identified by antiSMASH in the PT genome. However, we found that the T3PKS core gene of this BGC is also present in PT, with the same amino acid sequence. It is part of a BGC related to the one described for ochratoxin A (OTA) [28,29], and also reported in other *Fusarium* species (Figure 2). Indeed, as compared to the known OTA producers, a basic difference can be observed consisting in this gene replacing the NRPS gene (*otaB*), which is crucial for OTA biosynthesis. All the other genes of the 6-hydroxymellein BGC could be annotated, with some sequence differences between PT and Hzn5 relating to the T1PKS; in fact, in the latter strain this enzyme consists of 668 amino acids, while its length is more than 2500 amino acids in PT and the other *Fusarium* species considered in Figure 2. This truncation likely affects 6-hydroxymellein biosynthesis, as this metabolite was not detected in axenic cultures of Hzn5 [3].

As for culmorin and longiborneol (=juniperol), which were detected in the culture extracts of Hzn5 in our previous study [3], the gene *CLM1*, encoding for longiborneol synthase, was found by antiSMASH in an orphan BGC, i.e., a BGC having no similarity in the fungiSMASH database, in contig 16293 (antiSMASH region 40.8, Table 1) of the Hzn5 genome. Similarly, it could also be detected in the PT genome, showing a higher sequence identity (44.24% vs. 39.51% in Hzn5) with the corresponding amino acid sequences of a strain of *F. graminearum* (PH-1) [30]. However, antiSMASH was unable to find the gene encoding the cytochrome P450 monooxygenase (*CLM2*) catalyzing the hydroxylation of longiborneol to culmorin. Thus, we performed an additional search using the Exonerate software [31], which detected a similar gene in the same region, supporting the functionality of the orphan BGC. The putative *CLM2* sequence of Hzn5 is 1623 nt and 540 amino acids long and showed 29% similarity with the *F. graminearum* amino acid sequence used as query (GenBank: WXC54020.1).

### 2.5. Phylogenetic Relationships Referred to Selected Secondary Metabolites

Comparative analyses of genes encoding the synthesis of SMs provide an insight into their distribution across the genomes and the evolution of BGCs among *Fusarium* spp.; however, the distribution of BGCs in these fungi is not always strictly related to phylogenesis, considering the reported evidence of horizontal gene transfer (HGT) events [19].

Phylogenetic analyses were carried out examining the enzyme systems involved in the syntheses of enniatins, chrysogine, and fusarielins, representing the main SMs produced by isolate Hzn5 in axenic cultures, as documented in our previous study [3]. The analyses included the *Fusarium* species reported as producers of these compounds, as well as other non-*Fusarium* fungi sharing these biosynthetic abilities whose genome sequences are available in GenBank. Details concerning the selected strains are shown in Table S5.

**Figure 3:**
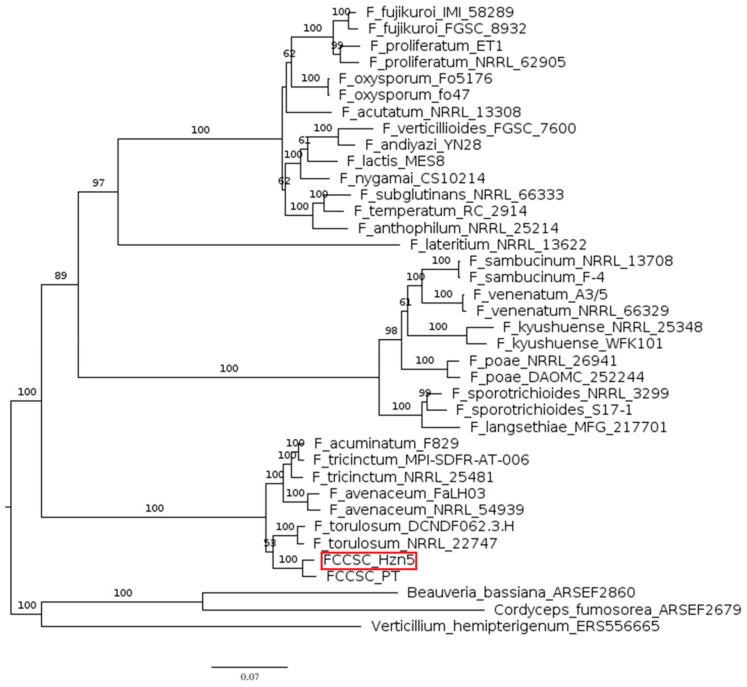
Phylogram including the known enniatin producers. The tree was based on maximum likelihood (ML) analysis of the enniatin synthetase amino acid sequence. Bootstrap support values ≥ 50% for ML are presented above branches. The longest branch of the unrooted tree was used as the outgroup. The scale bar indicates the number of nucleotide substitutions per site.

For enniatin production, we considered enniatin synthetase (esyn) from 23 *Fusarium* spp. and three additional Hypocreales species, namely *Beauveria bassiana, Cordyceps fumosorosea*, and *Verticillium hemipterigenum* (Figure 3). The latter are separated by notable phylogenetic distances, while the *Fusarium* spp. are grouped in three large clades; the hazelnut strains representing the FCCSC form an independent clade together with species in the FTSC, confirming their close relatedness.

**Figure 4:**
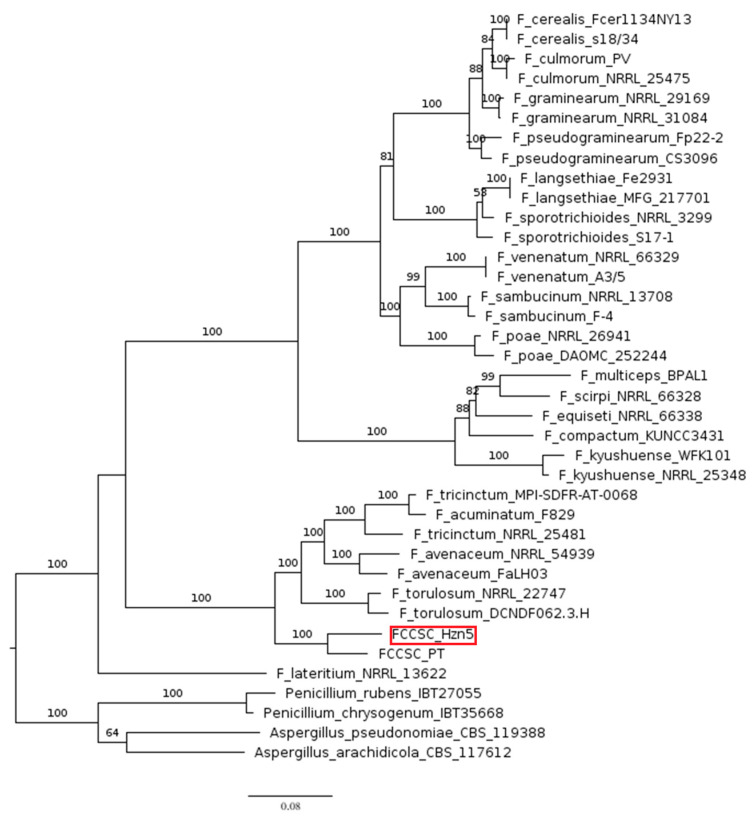
Phylogram including the known chrysogine producers. The tree was based on maximum likelihood (ML) analysis of the chrysogine synthase amino acid sequence. Bootstrap support values ≥ 50% for ML are presented above branches. The longest branch of the unrooted tree was used as the outgroup. The scale bar indicates the number of nucleotide substitutions per site.

Likewise, the clear proximity between species in the FTSC and the FCCSC results from the analysis based on chrysogine synthetase, including 19 *Fusarium* and a couple of *Penicillium* and *Aspergillus* species (Figure 4). Again, the latter are separated by a notable phylogenetic distance, while the *Fusarium* spp. are grouped in three distinct clades, one of which only includes the hazelnut strains and species in the FTSC. Interestingly, the only strain of *F. lateritium* whose genome sequence is available in GenBank is placed in an independent position in both the analyses.

**Figure 5:**
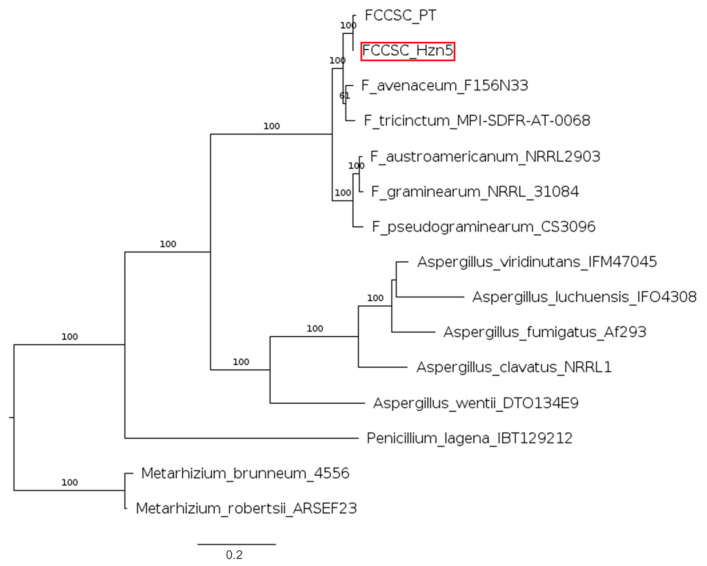
Phylogram including the known fusarielin producers. The tree was based on maximum likelihood (ML) analysis of the FSL1 PKS amino acid sequence. Bootstrap support values ≥ 50% for ML are presented above branches. The longest branch of the unrooted tree was used as the outgroup. The scale bar indicates the number of nucleotide substitutions per site.

Although infrequently reported, fusarielins are receiving increasing attention lately, with reference to both their occurrence and biological properties [32,33]. Besides a few *Fusarium* spp., production or biosynthetic potential for these decalin compounds has been reported from fungi in the genera *Aspergillus*, *Penicillium*, and *Metarhizium* [34,35]. The phylogenetic analysis concerning the main enzyme of the fusarielin BGC, that is, FSL1 polyketide synthase, again points out that the hazelnut isolates form a clade with two representatives of the FTSC, which is independent from the other three *Fusarium* producers (Figure 5). Interestingly, the phylogenetic distance from *Penicillium lagena* and five *Aspergillus* spp. (Eurotiomycetes, Eurotiales) is lower than from two *Metarhizium* spp., which otherwise are taxonomically closer as they are also classified in the Hypocreales.

## 3. Discussion

Although in our previous work microscopy evidenced some morphological differences between the hazelnut endophytic isolates and each of the five species so far described within the FCCSC, our phylogenetic reconstruction indicated that these isolates are closely related to the type strain of *F. celtidicola*. However, the phylogenetic distances among this species, *F. juglandicola*, and *F. aconidiale* may not justify their separation, calling for the examination of a larger set of isolates to support a more solid species typification. Isolate PT from symptomatic hazelnut also belongs to this major clade, indicating that its previous ascription to *F. tricinctum* [10] is incorrect. However, this conclusion is not surprising, considering that mismatch between *F. tricinctum* and *F. lateritium* has frequently occurred in the past [13], and that a recent examination of the strains of *F. lateritium* available in reputed collections has shown the majority of them to actually belong to the FCCSC [12]. Moreover, the latter study raised doubts on the distinction between *F. celtidicola* and *F. juglandicola*. It is to be expected that an upcoming phylogenetic reassessment of the FCCSC including all the *ex-lateritium* strains will conclusively define the taxonomic status of the hazelnut isolates.

Whatever the final response on the correct taxonomic placement, and considering the previous reports concerning *F. lateritium* from Italy and other countries where species identification is likely to have been mismatched [3], it is clear that this *Fusarium* sp. is of frequent occurrence on hazelnut as either endophyte or NCN agent, which raises concern about the possible mycotoxin contamination of kernels and derived products. The genome sequence of strain Hzn5, in comparison to the one available for isolate PT [10], enables to advance some considerations on their SM biosynthetic potential and on the possible toxicological risk deriving from their association with hazelnut.

**Figure 6:**
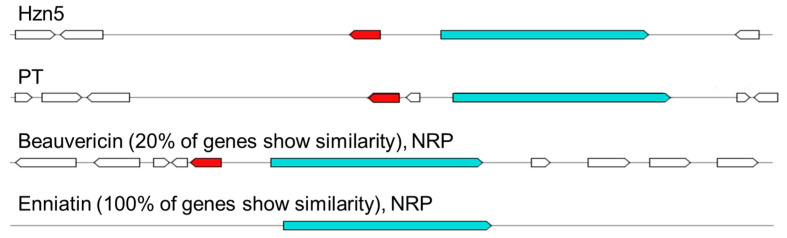
Comparison of the structure of the beauvericin BGC as resulting from antiSMASH known cluster comparison tab. Genes for enniatin synthetase and ketoisovalerate reductase are represented by a light blue and a red arrow, respectively. White arrows indicate other detected genes with unknown function in the context of enniatin synthesis.

Enniatins are cyclohexadepsipeptides synthesized through esyn, a multifunctional NRPS with a broad substrate specificity first characterized in *F. oxysporum* [36]. Indeed, esyn can accept different amino acids, explaining the variation in amino acid composition and the long list of enniatin analogues which have been identified up to now; the different affinity for amino acids is linked to differences in the sequence of the *esyn* gene, leading to peculiar enniatin profiles among the several producing species [37,38,39]. Enniatins are structurally homologous to beauvericin, which until recently was considered to differ in having phenylalanine as the N-methylated amino acid instead of leucine, isoleucine, or valine [40]. However, novel variants and hybrid compounds have been reported in the past few years, including the family of the beauvenniatins made of both aromatic and aliphatic amino acids, further complicating the analysis of the biochemical profile of *Fusarium* spp. [41,42]. Indeed, species in the FTSC were traditionally considered to only produce enniatins, until beauvericins were reported from *F. acuminatum, F. avenaceum*, and *F. tricinctum* as a result of more circumstantial studies [42,43]. However, this issue remains controversial, considering that the beauvericin BGC was not identified in the genome of the two latter species in a recent bioinformatic analysis [44]. Indeed, no beauvericins were found to be produced by both Hzn5 and the other conspecific isolate Hzn1 examined in our previous study [3]; the low similarity score resulting for beauvericin after the antiSMASH analysis (20%, corresponding to two out ten genes), shared with strain PT, is referable to esyn and 2-ketoisovalerate reductase, which are common to both biosynthetic pathways (Figure 6). In fact, despite the genomes of several enniatin-producing *Fusarium* species have been sequenced, no BGC specifically associated with the synthesis of these cyclohexadepsipeptides has been identified until recently [45].

Following assessments on their bioactivity, enniatins are regarded as emerging mycotoxins, and verification of their occurrence in food and feed following *Fusarium* infections in crop products has been recommended by the European Food Safety Authority [46,47]. Indeed, besides production in axenic culture, the synthesis of at least enniatin B by Hzn5 has been previously documented in planta in an experimental system [3]. Coupled with previous indications concerning the dried fruit sector [7], this evidence calls for further investigations on the possible contamination of hazelnut kernels and derived products.

Of course, similar considerations are valid for the other mycotoxins potentially produced by the hazelnut strains. It has been commonly verified that *Fusarium* isolates may not produce several SMs in axenic culture, despite holding the corresponding gene clusters [48]. Their biosynthetic potential could be expressed in planta or during product storage [49], following changes in the chromatin structure and the recruitment of transcription factors, or regulators, which are reported to affect biosynthesis of some SMs in *Fusarium* [50]. Moreover, the interaction with other microbial species which are part of the plant microbiome could unpredictably influence the synthesis and accumulation of mycotoxins in crop products.

In this respect, the presence of a genetic base and SMs which are related to the biosynthetic pathways of some important mycotoxins, such as 6-hydroxymellein and culmorin, should be more attentively considered in view of their possible impact on the safety of hazelnut products. It is known that 6-hydroxymellein is a precursor of terrein [51] and other fungal bioactive products [52]; conversely, despite the structural affinity, it is not directly involved in OTA biosynthesis [53]. Nevertheless, the presence in the 6-hydroxymellein BGC of a halogenase gene (*otaD*), encoding the last enzyme involved in OTA biosynthesis [29,53], combined with the gene gain/loss events in the OTA BGC reported among *Aspergillus* spp. [28], suggests further insights in the interconnections between the biosynthetic processes of these two products. Culmorin itself is regarded as an emerging mycotoxin, also influencing the toxicological properties of trichothecenes [54]. This sesquiterpenoid, first identified as a product of *F. culmorum*, is synthesized starting from farnesyl diphosphate, which is converted to longiborneol by the *CLM1*-encoded synthase; the latter compound is finally hydroxylated by the cytochrome P450 monooxygenase encoded by *CLM2* [55]. Longiborneol is a known SM of *F. tricinctum* and related species [56], while culmorin has never been reported in the FTSC [13,56]. However, the presence of the above biosynthetic genes has been documented in the genome of several representatives of this species complex, namely *F. acuminatum, F. avenaceum, F. torulosum*, and *F. tricinctum* [54], which is in line with our findings concerning the hazelnut strains. *CLM1* and *CLM2* are part of a BGC which is labeled as culmorin/(+)-juniperol(longiborneol)/15-acetyldeoxynivalenol in antiSMASH. As typically observed in *F. graminearum* and some related species in the *F. sambucinum* species complex [57], this BGC includes the *TRI* genes responsible for the synthesis of trichothecenes. In addition to their bad reputation as mycotoxins, these products are well-known determinants of pathogenicity in *Fusarium* [58]; notwithstanding, no *TRI* genes were found in association to *CLM*s in both PT and Hzn5, which is corroborated by the observed lack of trichothecene production in axenic culture of the latter [3]. However, it cannot be disregarded that the biosynthetic conditions in vivo or during storage could bring to the production of these mycotoxins to some extent. Besides the toxicological implications, the moderate antifungal activity and the low insecticidal effects characterizing culmorin, coupled with those documented for the enniatins, chrysogine, and the fusarielins [3], call for a possible role of these *Fusarium* associates as defensive mutualists of hazelnut.

Fungal chemodiversity basically stems from three evolutionary mechanisms involving BGCs: functional divergence, HGT, and de novo assembly [59]. Particularly, HGT may even involve whole chromosomes, as it has been shown in *Fusarium poae* where apicidin biosynthesis is linked to isolate-specific putative accessory chromosomes [60]. The integration with accessory chromosomes may provide plant-associated strains with additional biosynthetic potential and eventually determine their transformation into pathogens [61]. While the BGCs which are widely conserved among *Fusarium* species (e.g., fusarubins, gibepyrone A) represent an indication for their ancestral presence in the genome of these fungi, the absence/presence of other BGCs encoding for infrequent compounds may depend on either a complete or partial loss during the evolution of certain lineages, or a take-over from other ecologically associated microorganisms through HGT [62]. For instance, phylogenetic analyses have pointed out that the introduction of several NRPS and PKS genes from other *Fusarium* lineages through HGT is responsible for the irregular distribution patterns of SMs among species within the *Fusarium incarnatum-equiseti* species complex [63]. In our phylogenetic analyses considering the key enzymes involved in the synthesis of the main SMs produced by Hzn5 in axenic cultures, the distances between *Fusarium*- and non-*Fusarium*-producing species are indicative that the related genomic differences may depend on ordinary evolutionary divergence rather than HGT from other fungi.

## 4. Materials and Methods

### 4.1. Morphological Observations

A 5 mm mycelial disc from the edge of an actively developing single-spore culture of strain Hzn5 was positioned at the center of fresh PDA (Difco, Detroit, MI, USA) and synthetic nutrient agar (SNA, made from ingredients in the laboratory) plates, which were incubated in the dark at 25 ± 1 °C. After 10 days on PDA, observations of the culture phenotype were recorded considering colony diameter, front and reverse color, margins, pigment production, and general appearance. Micromorphological features (i.e., macroconidia, microconidia, chlamydospores, and conidiogenous cells) were examined using a light microscope equipped with a 1 MP Motic camera (Nikon Eclipse Ni-U, Tokyo, Japan). Measurements of conidia were taken and analyzed using the JMicro Vision v.1.3.4 program and ScopeImage 9.0 software (Bioimager, Vaughan, Canada).

### 4.2. Phylogenetic Analysis

A phylogenetic analysis considering the *Fusarium* species/strains listed in Table S1 was carried out based on the concatemers of the two most relevant genetic markers used for species identification within the FCCSC (*tef1* and *rpb2*) [11]. The combined *tef1* and *rpb2* sequences were aligned using Muscle ver. 3.8 [64] and phylogenetic analyses of the concatenated sequence data for maximum likelihood (ML) were performed using RAxML software version 8.2.12 [65] with 1000 bootstrap replications. The phylogenetic tree was drawn using the software FigTree v1.4.3.

### 4.3. Genome Sequencing and Assembly

#### 4.3.1. Starting Material, DNA Extraction, and Sequencing

A liquid culture in potato dextrose broth (PDB, 100 mL) was initiated by inoculating a 5 mm mycelial disc from the edge of an actively developing PDA culture of isolate Hzn5, which was grown in darkness at 25 °C. After 7 days, the mycelium was collected, lyophilized, and stored at −20 °C. To obtain high-quality and high-molecular-weight genomic DNA for next generation sequencing (NGS), about 10 mg of lyophilized mycelium was finely ground at dry-ice temperature through stainless steel beads in 2 mL Eppendorf tubes using a TissueLyser apparatus (Qiagen S.r.l., Milano, Italy), twice for 1 min at 30 Hz. Then, 1 mL of lysis buffer of the GeneJET Plant Genomic DNA Purification Mini Kit (Thermo Fisher Scientific, Waltham, MA, USA) was added. DNA was extracted following the manufacturer’s indications utilizing spin columns with silica-based membrane technology. After the final elution steps, purity and quality of DNA were checked on 2 μL of sample by the 260/280 and 260/230 nm absorbance ratios in a UV-Vis spectrophotometer (NanoDrop ND-1000, Thermo Fisher Scientific). DNA was quantified by absorbance at 260 nm, and its integrity was assessed by agarose (1.5% *w*/*v*) gel electrophoresis. Finally, a 600 ng aliquot of the genomic DNA was brought to a volume of 30 µL (20 ng µL^−1^) by a Concentrator 5301 centrifuge (Eppendorf S.r.l., Milano, Italy) and submitted to NGS. Whole genome sequencing was performed according to the manufacturer’s indications using the NovaSeq6000 Sequencing System (Illumina Inc., San Diego, CA, USA) with a paired-end sequencing (2 × 150 bp), obtaining 59,076,306 paired reads. Library preparation, sequencing, and bioinformatics analysis were performed by Genomix4Life (Baronissi, Salerno, Italy).

#### 4.3.2. Genome Data Processing: De Novo Assembly and Annotation

Prior to further analysis, a quality check of the reads was performed on the genome sequencing data by the FastQC tool (Babraham Bioinformatics, Cambridge, UK). The FastQC tool returns an html report in which information about raw sequencing data can be visualized through a summary judgment. The best k-mer length was estimated by using the Velvet Advisor tool [66] attempting to optimize k by a Velvet assembly for each odd k-value picking the one that yields the highest scaffold N50. Then, the genome de novo assembly was performed by ABySS 2.0 [67], an implementation of ABySS 1.0 [68], that uses a multi-stage pipeline consisting of unitig, contig, and scaffold stages. ABySS 2.0 follows the model of Minia, wherein a probabilistic Bloom filter representation is used to encode the de Bruijn graph, which is a directed graph representing overlaps between sequences of m symbols. In the context of Bloom filter-based de Bruijn graph assembly algorithms, the elements of the set are the k-mers of the input sequencing reads.

To evaluate genome assembly quality and completeness, we used QUAST and BUSCO tools, respectively [14,15]. QUAST determines the maximum contig length for the genome and the maximum scaffold length with N50, as well as the total length of the contigs for scaffolds. BUSCO (v. 5.7.1) is based on the concept of single-copy orthologs that should be highly conserved among closely related species; in this analysis we used single-copy orthologs discovered among the Hypocreales. The lineage dataset used was hypocreales_odb10 (creation date: 2024-01-08, number of genomes: 50, number of BUSCOs: 4494). BUSCO’s results are simplified into categories. BLASTn was used to perform the annotation of the scaffolds [16]. This tool compares one or more nucleotide query sequences to a subject nucleotide sequence or a database of nucleotide sequences. In this study, the subject database included all the nucleotide sequences belonging to the Fungi kingdom. Simultaneously, we conducted a genome annotation using BUSCO’s mapping algorithm (Miniprot) that uses a reference protein database (provided in the BUSCO datasets) to map proteins to the genome. In this case, the reference dataset contains the protein sequences of the fungal species belonging to the Hypocreales. The closest dataset in phylogenetic terms is chosen automatically by the software. The result of this mapping allowed the detection of different genes in the assembly. Subsequently, a functional analysis was carried out via the OmicsBox software ver. 3.2 that contains several tools for this purpose. First, starting from the BLASTn alignments previously obtained, the InterPro tool was used to search for protein families and predict domains and important amino acidic sites. Then, we performed an analysis of the pathways (KEGG and reactome pathway databases) in which predicted genes were involved.

#### 4.3.3. Biosynthetic Gene Clusters Prediction and Analysis

The main SM-BGCs in Hzn5 were identified using the antiSMASH database, fungal version 7.1 (fungiSMASH), with the default settings [17]. Moreover, this tool was used to comparatively investigate the genome of isolate PT [10].

A phylogenetic reconstruction for the key enzymes involved in enniatins, chrysogine, and fusarielins biosynthesis was conducted. Briefly, the amino acid sequences of the key biosynthetic enzymes were retrieved from fungiSMASH results and used as queries in BLAST searches to retrieve homologous sequences in the NCBI RefSeq Reference Genome database. The homology was confirmed using the tBLASTn results as input in fungiSMASH. The sequences were considered homologous when the same BGC annotation was obtained. After this validation step, homologous amino acid sequences were aligned using Muscle ver. 3.8 [64], with default settings. Alignments were automatically trimmed using Gblocks version 0.91b [69] to avoid comparisons of non-conserved regions present only in a subset of the taxa. The best-fit model of amino acid substitution and phylogenetic reconstruction was generated using RAxML 8.2.12 [65]. The maximum likelihood tree was run for 1000 bootstrap replicates, and the tree figure was plotted using FigTree v1.4.3.

Additional analyses to assess the presence/absence of specific BGC member genes were conducted by scanning the Hzn5 and PT genomes using the Exonerate software [31]. In brief, the amino acid sequences of CLM2 (from *F. graminearum* KSU23473, GenBank: WXC54020) and TRI biosynthetic enzymes (from *Fusarium* sp. NRRL 1345, GenBank: GQ865563.1) were used as inputs in Exonerate using the protein2genome model; it allows introns in the alignment, but also frameshifts, and exon phase changes when a codon is split by an intron.

## 5. Conclusions

The complete genome sequence of the hazelnut endophytic isolate Hzn5 from Poland belonging to the FCCSC is reported with this work, revealing a close phylogenetic relationship with a hazelnut pathogenic isolate from Italy previously ascribed to *F. tricinctum*. The SM-BGC profiles of the examined isolates are quite similar and confirm the taxonomic proximity to the FTSC. The ascertained production of enniatins, coupled with the potential for SMs related to the biosynthetic pathways of other mycotoxins, raises some concern for the possible contamination of hazelnuts and derived products, which deserves greater attention by the stakeholders.

## Figures and Tables

**Table 1:** BGCs identified by antiSMASH in the Hzn5 genome and their similarity scores in comparison to strain PT.

Contig	Region	Type	From	To	Most Similar Known BGCs	Similarity (%)	
						Hzn5	PT
16265	18.1	NRPS	64,724	127,601			
16275	22.1	T1PKS	754,845	804,388	orcinol/orsellinic acid	55	55
16279	23.1	NRPS-like, T1PKS	137,159	195,811	fusarielin H	62	50
16279	23.2	terpene	325,453	348,561	gibberellin	28	42
16265	33.1	betalactone	270,136	303,018			
16265	33.2	NRPS-like	667,535	711,386	choline	100	100
16265	33.3	NRPS-like	787,157	832,949			
16265	33.4	T1PKS, NRPS	979,234	1,031,349	fusaridione A	18	18
16257	37.1	T1PKS, NRPS	25,277	136,354			
16257	37.2	T3PKS	382,522	423,836	6-hydroxymellein	33	nd
16257	37.3	NRPS-like	470,544	513,917			
16257	37.4	T1PKS, NRPS	1,879,135	1,931,246	ilicicolin H,J/8-epi-ilicicolin H	60	60
16257	37.5	fungal-RiPP-like	2,346,099	2,409,176			
16277	39.1	T1PKS	1	40,460	fujikurins A−D	50	83
16277	40.1	NRPS	119,297	175,596			
16277	40.2	terpene	247,516	268,206			
16277	40.3	NRPS-like	444,480	488,237			
16277	40.4	NRPS	1,135,033	1,228,604			
16277	40.5	fungal-RiPP-like	1,534,027	1,594,923			
16293	40.6	T1PKS	1,617,087	1,665,688	gibepyrone A	40	40
16293	40.7	NRPS-like	3,606,132	3,649,956	bassianolide	13	13
16293	40.8	terpene	4,308,961	4,329,877	*CLM1, CLM2* ^1^		
16283	40.9	NRPS	4,372,229	4,420,244	chrysogine	83	83
16283	40.10	NRPS	4,435,111	4,504,467			
16248	42.1	NRPS, T1PKS	40,368	103,235	fusaristatin A	100	80
16247	47.1	phosphonate	275,063	296,779	fosfonochlorin	53	61
16247	47.2	T1PKS	376,594	423,598	bikaverin	42	42
16247	47.3	T3PKS	978,133	1,019,603			
16247	47.4	T1PKS	2,304,527	2,352,015			
16297	49.1	fungal-RiPP-like	137,098	198,224			
16290	49.2	NRPS, T1PKS	404,613	456,534	ACT-toxin II	100	100
16290	49.3	T1PKS	570,192	616,346	bikaverin	57	57
16276	50.1	T1PKS	364,207	411,510	oxyjavanicin	62	50
16276	50.2	terpene	1,034,967	1,056,515	squalestatin S1	40	40
16276	50.3	NRP-metallophore, NRPS	2,607,962	2,671,361			
16303	51.1	T1PKS	1	28,837			
16303	51.2	NRPS-like	234,494	277,357			
16303	51.3	terpene	554,993	579,071			
16303	51.4	terpene	670,601	691,870	α-acorenol	100	100
16303	51.5	NRPS	1,179,738	1,228,091			
16320	52.1	terpene	323,302	345,735			
16319	52.2	T1PKS	2,037,563	2,085,451			
16320	53.1	T1PKS	97,452	147,762			
16320	53.2	fungal-RiPP-like, T1PKS	148,668	215,872	fusarubin/1233A–B/NG-391/lucilactaene	28	nd
16320	53.3	NRPS	592,686	640,553			
16254	54.1	terpene	719,300	741,312			
16254	54.2	terpene	1,138,511	1,160,268	koraiol	100	100
16254	54.3	fungal-RiPP-like, isocyanide	1,269,111	1,347,781			
16321	55.1	NRPS	268,291	317,683	beauvericin ^2^	20	20

^1^ The genes *CLM1* and *CLM2* were found to be part of this orphan BGC. ^2^ This BGC includes enniatin synthetase. nd = not detected.

## Data Availability

Raw data and output files, including sequences, alignments, and phylogenetic trees are available from Zenodo repository at https://doi.org/10.5281/zenodo.15082681.

## Supplementary Materials

The following supporting information can be downloaded at https://www.mdpi.com/article/10.3390/ijms26094377/s1.
