# Supplementary material for: Genome Sequencing of a Fusarium Endophytic Isolate from Hazelnut: Phylogenetic and Metabolomic Implications
Source: Int J Mol Sci. 2025 May 5;26(9):4377. doi: 10.3390/ijms26094377 (PMC12072968; doi:10.3390/ijms26094377)
Supplement: Supplementary file 1 [file ijms-26-04377-s001.zip › Figure S1. Hzn5 culture.pdf]

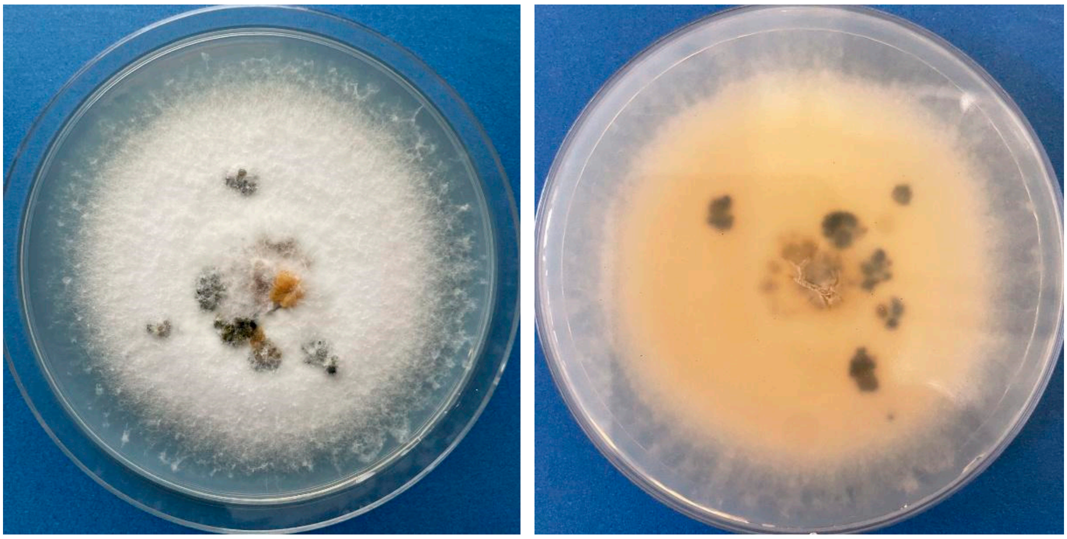

Figure S1. Thirty-day-old culture on PDA of *Fusarium* isolate Hzn5, showing formation of sclerotia on the mycelium surface (left) and absence of pigmentation at the reverse (right).
