# Supplementary material for: Genome Sequencing of a Fusarium Endophytic Isolate from Hazelnut: Phylogenetic and Metabolomic Implications
Source: Int J Mol Sci. 2025 May 5;26(9):4377. doi: 10.3390/ijms26094377 (PMC12072968; doi:10.3390/ijms26094377)
Supplement: Supplementary file 1 [file ijms-26-04377-s001.zip › Figure S2. Sequence quality modules.pdf]

Per base sequence quality

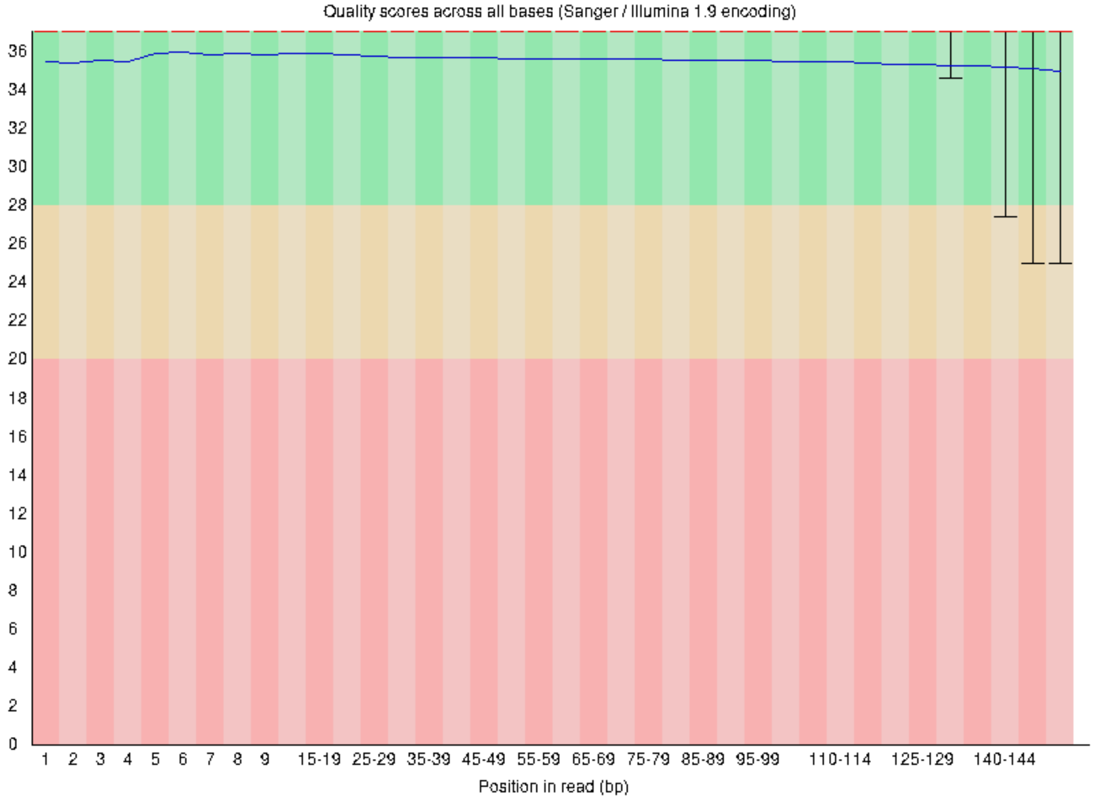

Per base sequence quality

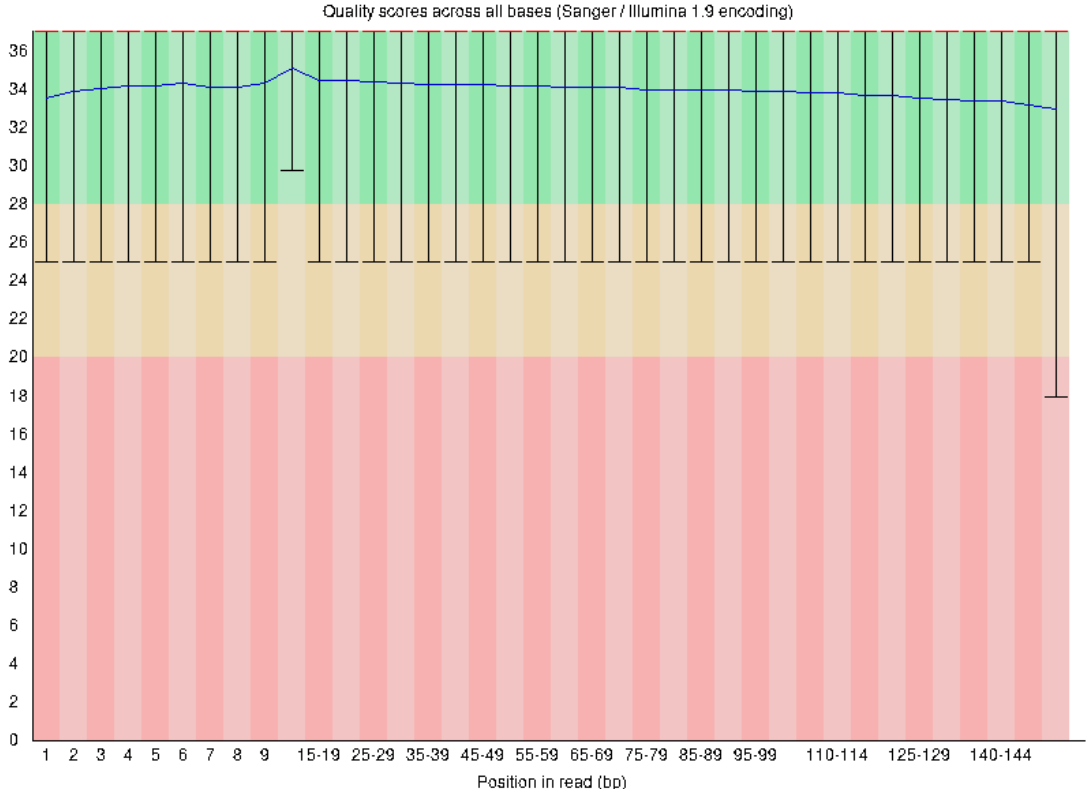

Figure S2. Per base sequence quality modules of the isolate Hzn5 in both forward (R1, above) and reverse (R2, below) fastq file.
