# Supplementary material for: Genome Sequencing of a Fusarium Endophytic Isolate from Hazelnut: Phylogenetic and Metabolomic Implications
Source: Int J Mol Sci. 2025 May 5;26(9):4377. doi: 10.3390/ijms26094377 (PMC12072968; doi:10.3390/ijms26094377)
Supplement: Supplementary file 1 [file ijms-26-04377-s001.zip › Figure S3A. Quality assessment for genome assembly.pdf]

QUAST

Quality Assessment Tool for Genome Assemblies by CAB

25 June 2024, Tuesday, 09:59:53

View in Icarus contig browser

All statistics are based on contigs of size >= 500 bp, unless otherwise noted (e.g., "# contigs (>= 0 bp)" and "Total length (>= 0 bp)" include all contigs)

Statistics without reference	fusarium_contigs
# contigs	187
# contigs (>= 0 bp)	10 271
# contigs (>= 1000 bp)	100
# contigs (>= 5000 bp)	77
# contigs (>= 10000 bp)	67
# contigs (>= 25000 bp)	62
# contigs (>= 50000 bp)	61
Largest contig	1 776 231
Total length	41 543 006
Total length (>= 0 bp)	43 287 613
Total length (>= 1000 bp)	41 486 149
Total length (>= 5000 bp)	41 441 506
Total length (>= 10000 bp)	41 374 531
Total length (>= 25000 bp)	41 301 515
Total length (>= 50000 bp)	41 260 744
N50	1 148 930
N75	609 833
L50	15
L75	26
GC (%)	47.34
**Mismatches**	
# N's	908
# N's per 100 kbp	2.19

Plots: Cumulative length Nx GC content

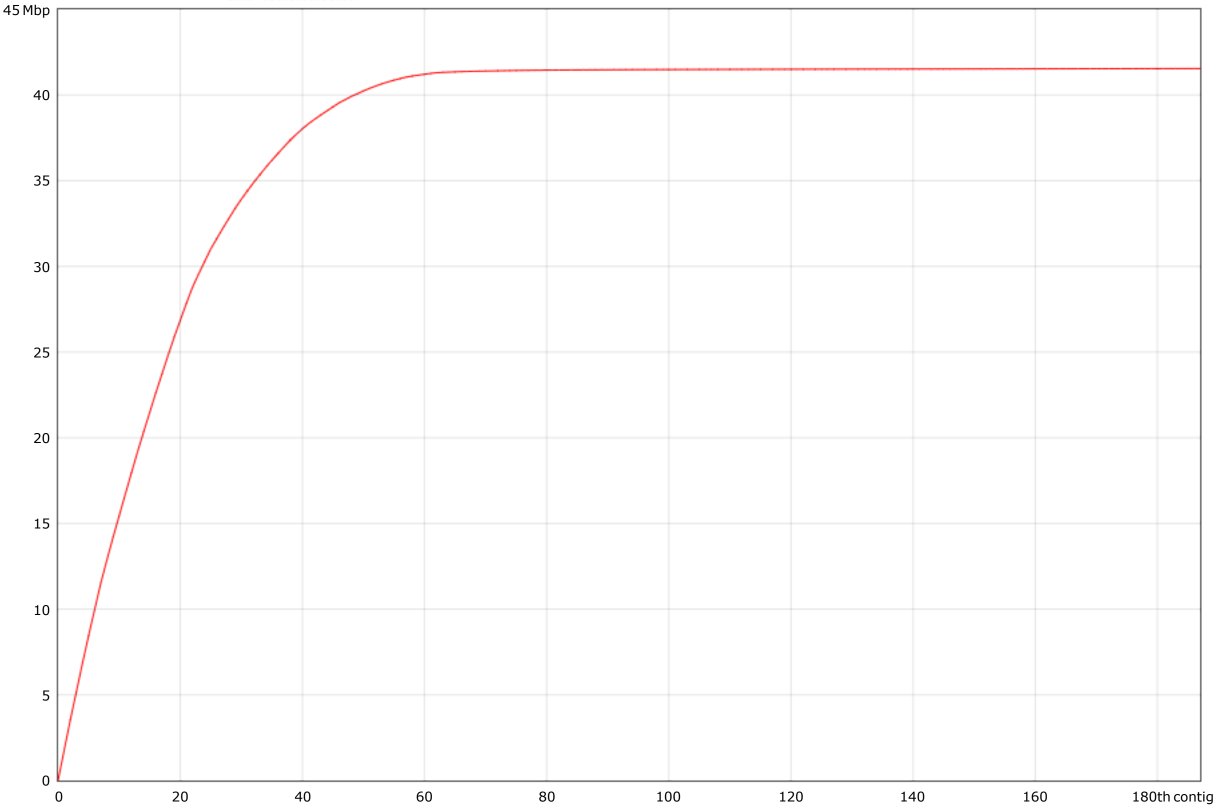

Contigs are ordered from largest (contig #1) to smallest.
