# Supplementary material for: Genome Sequencing of a Fusarium Endophytic Isolate from Hazelnut: Phylogenetic and Metabolomic Implications
Source: Int J Mol Sci. 2025 May 5;26(9):4377. doi: 10.3390/ijms26094377 (PMC12072968; doi:10.3390/ijms26094377)
Supplement: Supplementary file 1 [file ijms-26-04377-s001.zip › Figure S3B. Quality assessment for genome assembly.pdf]

QUAST

Quality Assessment Tool for Genome Assemblies by CAB

08 July 2024, Monday, 11:25:38

View in Icarus contig browser

All statistics are based on contigs of size >= 500 bp, unless otherwise noted (e.g., "# contigs (>= 0 bp)" and "Total length (>= 0 bp)" include all contigs)

Statistics without reference	fusarium_scaffolds
# contigs	140
# contigs (>= 0 bp)	10 185
# contigs (>= 1000 bp)	55
# contigs (>= 5000 bp)	39
# contigs (>= 10000 bp)	31
# contigs (>= 25000 bp)	26
# contigs (>= 50000 bp)	26
Largest contig	5 912 375
Total length	41 544 408
Total length (>= 0 bp)	43 279 897
Total length (>= 1000 bp)	41 488 799
Total length (>= 5000 bp)	41 459 193
Total length (>= 10000 bp)	41 404 077
Total length (>= 25000 bp)	41 323 722
Total length (>= 50000 bp)	41 323 722
N50	2 818 754
N75	1 580 901
L50	5
L75	10
GC (%)	47.34
**Mismatches**	
# N's	1582
# N's per 100 kbp	3.81

Plots: Cumulative length Nx GC content

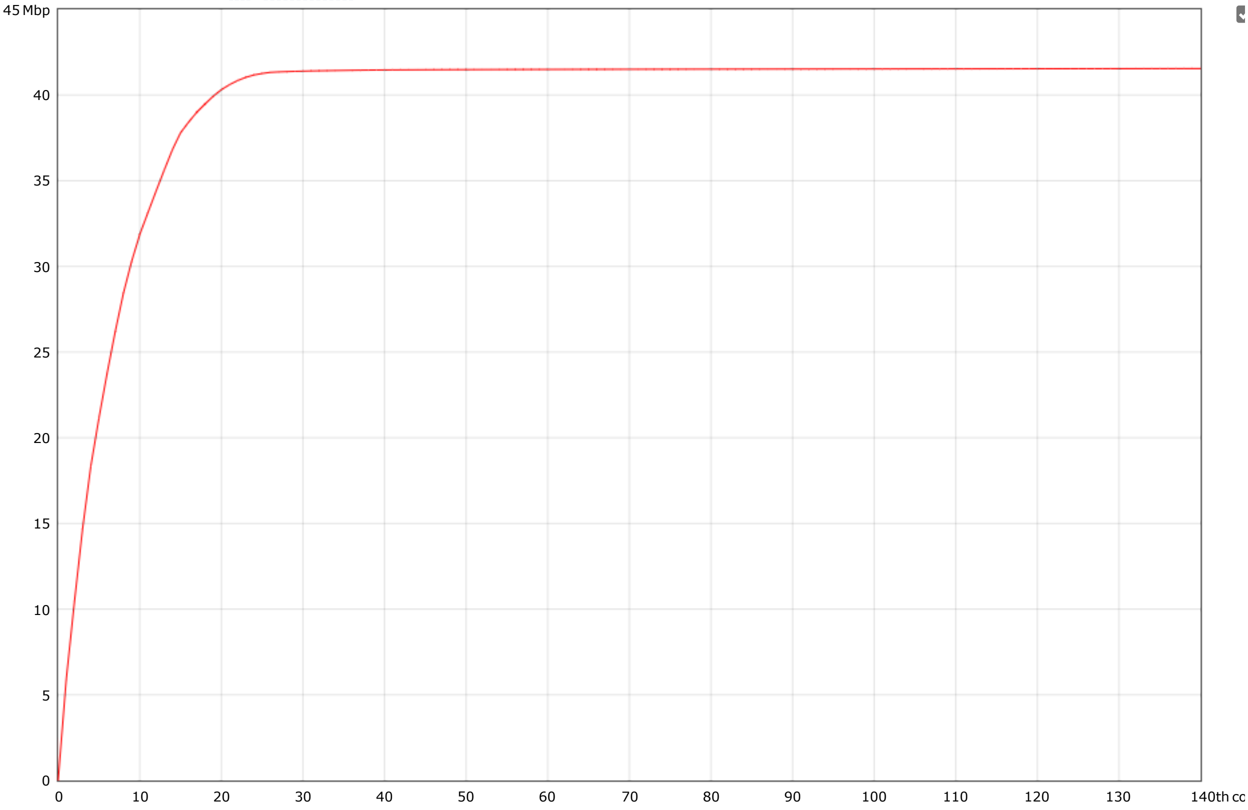

Contigs are ordered from largest (contig #1) to smallest.
