# Supplementary material for: Genome Sequencing of a Fusarium Endophytic Isolate from Hazelnut: Phylogenetic and Metabolomic Implications
Source: Int J Mol Sci. 2025 May 5;26(9):4377. doi: 10.3390/ijms26094377 (PMC12072968; doi:10.3390/ijms26094377)
Supplement: Supplementary file 1 [file ijms-26-04377-s001.zip › Table S1. GenBank codes.pdf]

Table S1. GenBank codes of *tef1* and *rpb2* sequences of the strains considered in the phylogenetic analysis.

***Fusarium* species**	**Strain number**	**Origin**	***tef1***	***rpb2***
F. aconidiale	UBOCC-A-109005	*Triticum aestivum*, France	MZ078246	MZ078218
F. acuminatum	NRRL 45994	cloaca, USA	GQ505432	GQ505496
F. avenaceum	NRRL 39591	garden pea, New Zealand	OL772757	OL773061
F. celtidicola	MFLUCC 16-0526	*Celtis australis*, Italy	ON745620	ON759296
F. citricola	CPC 27067	*Citrus limon*, Italy	LT746194	LT746307
F. citricola	CPC 27069	*Citrus sinensis*, Italy	LT746195	LT746308
F. citricola	CPC 27709	*C. sinensis*, Italy	LT746196	LT746309
F. citricola	CPC 27805	*Citrus reticulata*, Italy	LT746197	LT746310
F. citricola	CPC 27813	*C. reticulata*, Italy	LT746198	LT746311
F. flocciferum	NRRL 45999	human scalp, USA	GQ505433	GQ505497
F. gamsii	NRRL 34036	human ethmoid sinus, USA	GQ505419	GQ505483
F. iranicum	NRRL 52714	*Eurygaster* sp., Turkey	JF740796	JF740977
F. juglandicola	UBOCC-A-101147	*Juglans regia*, France	MZ078244	MZ078216
F. juglandicola	UBOCC-A-102014	*J. regia*, France	MZ078245	MZ078217
F. juglandicola	UBOCC-A-119001	*J. regia*, France	MZ078243	MZ078215
F. lateritium	NRRL 13622	*Ulmus* sp., USA	PQ000518	JX171571
F. negundinis	NRRL 28287	polypore, USA	OL772754	OL773058
F. reticulatum	NRRL 25792	soil, Denmark	OL772846	OL773150
F. salinense	CPC 26403	*C. sinensis*, Italy	LT746191	LT746304
F. salinense	CPC 26457	*C. sinensis*, Italy	LT746192	LT746305
F. salinense	CPC 26973	*C. sinensis*, Italy	LT746193	LT746306
F. sinensis	KOD 941	wheat seed, China	OL772795	OL773099
F. torulosum	NRRL 52772	*Galleria mellonella* larva, Norway	MH582397	JF740804
F. tricinctum	F1544	*Triticum durum*, Italy	OL964791	OL658768
F. tricinctum	NRRL 25481	winter wheat, Germany	OL772833	MH582357
FCCSC	Hzn1	*Corylus avellana* endophyte, Poland	PP788632	PP738981
FCCSC	Hzn5	*C. avellana* endophyte, Poland	PP788631	PP738982
FCCSC	IHEM 28077	bat, Belgium	OU641411	OU641410
FCCSC	PT	*C. avellana* necrotic kernel, Italy	GCA_021020635.1 *	
FCCSC	ZLVG.982	*Pinus sylvestris*, Slovenia	OR105858	OR098304
FTSC sp. 5	NRRL 53679	*Quercus robur* leaf, Netherlands	OL772766	OL773070
FTSC sp. 8	NRRL 52722	*Eurygaster* sp., Turkey	JF740804	JF740980
FTSC sp. 10	NRRL 36132	*Peltigera rufescens*, Netherlands	OL772789	OL773093
FTSC sp. 11	NRRL A-28042	spruce seedling, USA	OL772761	OL773065
FTSC sp. 12	NRRL 40096	alfalfa, New Zealand	OL772839	OL773143
FTSC sp. 15	P78c	*T. durum*, Italy	OL964788	OL658810
FTSC sp. 17	NRRL 53952	unknown	OL772853	OL773157
FTSC sp. 18	NRRL 36565	unknown	OL772876	OL773180
FTSC sp. 19	NRRL A-28022	soil, Australia	OL772778	OL773082
FTSC sp. 20	FRC R-7848	apple, Australia	OL772774	OL773078
FTSC sp. 21	NRRL 39814	blackberry, New Zealand	OL772784	OL773088
FTSC sp. 22	FRC R-6739	codling moth, Germany	OL772764	OL773068
FTSC sp. 23	FRC R-7739	peat moss, USA	OL772835	OL773139
FTSC sp. 24	FRC R-7356	soil, China	OL772894	OL773198
FTSC sp. 25	FRC R-4587	soil, Australia	OL772866	OL773170

* Marker sequences extracted from the genome.
