# Supplementary material for: Genome Sequencing of a Fusarium Endophytic Isolate from Hazelnut: Phylogenetic and Metabolomic Implications
Source: Int J Mol Sci. 2025 May 5;26(9):4377. doi: 10.3390/ijms26094377 (PMC12072968; doi:10.3390/ijms26094377)
Supplement: Supplementary file 1 [file ijms-26-04377-s001.zip › Table S5. Fungal strains and genome codes.pdf]

Table S5. List of the fungal strains and genome codes used for the phylogenetic analyses referring to the core genes in the BGCs of the main secondary metabolites identified in Hzn5.

Species	Strain number	Gene	Genome code
Aspergillus arachidicola	CBS 117612	C	GCA_009193545.1
Aspergillus clavatus	NRRL 1	F	GCA_000002715.1
Aspergillus fumigatus	Af293	F	GCA_000002655.1
Aspergillus luchuensis	IFO4308	F	GCA_016861625.1
Aspergillus pseudonomiae	CBS 119388	C	GCA_009193645.1
Aspergillus viridinutans	IFM 47045	F	GCA_018404265.1
Aspergillus wentii	DTO 134E9	F	GCA_001890725.1
Beauveria bassiana	ARSEF2860	E	GCF_000280675.1
Cordyceps fumosorosea	ARSEF2679	E	GCA_001636725.1
FCCSC	Hzn5	C, E, F	SRR31988993
FCCSC	PT	C, E, F	GCA_021020635.1
Fusarium acuminatum	F829	C, E	GCA_013363215.1
Fusarium acutatum	NRRL 13308	E	GCA_012932015.1
Fusarium andiyazi	YN28	E	GCA_044706615.1
Fusarium anthophilum	NRRL 25214	E	GCA_013364935.1
Fusarium austroamericanum	NRRL 2903	F	GCA_013364965.1
Fusarium avenaceum	F156N33	F	GCA_018282135.1
F. avenaceum	NRRL 54939	C, E	GCA_000769215.1
F. avenaceum	FaLH03	C, E	GCA_000769305.1
Fusarium cerealis	Fcer1134NY13	C	GCA_012600195.1
F. cerealis	S18/34	C	GCA_019055205.1
Fusarium compactum	KUNCC3431	C	GCA_037577415.1
Fusarium culmorum	NRRL 25475	C	GCA_013618375.1
F. culmorum	PV	C	GCA_003033665.1
Fusarium equiseti	NRRL 66338	C	GCA_004367125.1
Fusarium fujikuroi	FGSC 8932	E	GCA_001023045.1
F. fujikuroi	IMI 58289	E	GCA_900079805.1
Fusarium graminearum	NRRL 29169	C	GCA_023242275.1
F. graminearum	NRRL 31084 (PH1)	C, F	GCA_000240135.3
Fusarium kyushuense	NRRL 25348	C, E	GCA_013184315.1
F. kyushuense	WFK191	C, E	GCA_034753255.1
Fusarium lactis	MES8	E	GCA_044647025.1
Fusarium langsethiae	Fe2931	C	GCA_018104275.1
F. langsethiae	MFG 217701	C, E	GCA_022180325.1
Fusarium lateritium	NRRL 13622	C, E	GCA_014898835.1
Fusarium multiceps	BPAL1	C	GCA_028416715.1
Fusarium nygamai	CS10214	E	GCA_002894225.1
Fusarium oxysporum	Fo47	E	GCF_013085055.1
F. oxysporum	Fo5176	E	GCA_030345115.2
Fusarium poae	DAOMC 252244	C, E	GCA_019609905.1
F. poae	NRRL 26941	C, E	GCA_013623615.1
Fusarium proliferatum	ET1	E	GCF_900067095.1
F. proliferatum	NRRL 62905	E	GCA_036288945.1
Fusarium pseudograminearum	CS3096	C, F	GCA_000303195.2
F. pseudograminearum	Fp22-2	C	GCA_000303195.2
Fusarium sambucinum	F-4	C, E	GCA_001567575.1
F. sambucinum	NRRL 13708	C, E	GCA_014899025.1
Fusarium scirpi	NRRL 66328	C	GCA_004367495.1
Fusarium sporotrichioides	NRRL 3299	C, E	GCA_003012315.1
F. sporotrichioides	S17/1	C, E	GCA_019054675.1
Fusarium subglutinans	NRRL 66333	E	GCA_013396075.1
Fusarium temperatum	RC 2914	E	GCA_012070365.1
Fusarium torulosum	DCNDF062.3.H	C, E	GCA_044647435.1
F. torulosum	NRRL 22747	C, E	GCA_013623875.1
Fusarium tricinctum	MPI-SDFR-AT-0068	C, E, F	GCA_020744515.1

F. tricinctum	NRRL 25481	C, E	GCA_012977725.1
Fusarium venenatum	A3/5	C, E	GCA_019425555.1
F. venenatum	NRRL 66329	C, E	GCA_013623635.1
Fusarium verticillioides	FGSC 7600	E	GCA_000149555.1
Metarhizium brunneum	4556	F	GCA_013426205.1
Metarhizium robertsii	ARSEF23	F	GCA_000187425.2
Penicillium chrysogenum	IBT 35668	C	GCA_028827035.1
Penicillium lagena	IBT 129212	F	GCA_028827675.1
Penicillium rubens	IBT 27055	C	GCA_028828025.1
Verticillium hemipterigenum	ERS556665	E	GCA_000825705.1

C = chrysogine synthetase; E = enniatin synthetase; F = FSL1 polyketide synthase.
