# Supplementary figures and images for: Genome Sequencing of a Fusarium Endophytic Isolate from Hazelnut: Phylogenetic and Metabolomic Implications
Source: Int J Mol Sci. 2025 May 5;26(9):4377. doi: 10.3390/ijms26094377 (PMC12072968; doi:10.3390/ijms26094377)

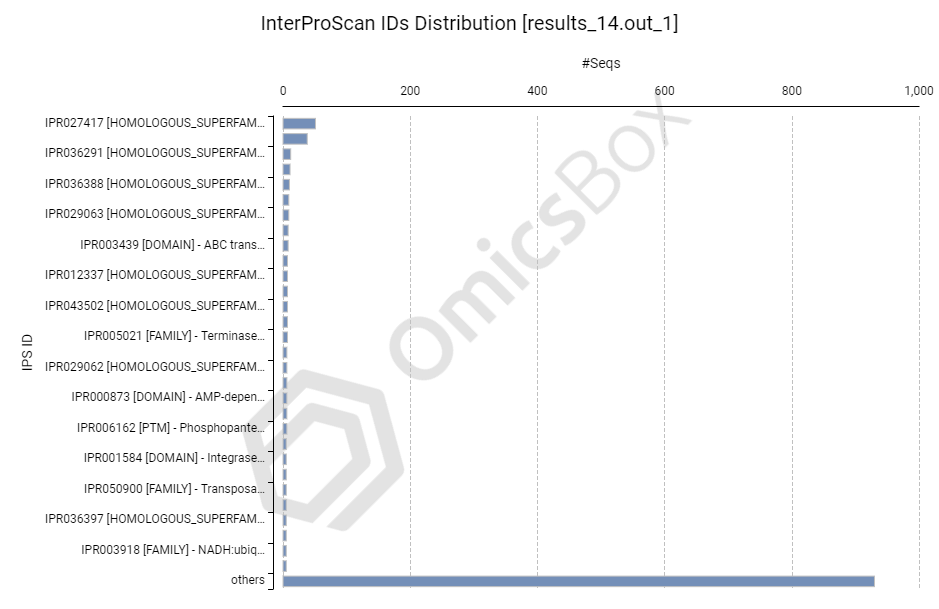

Supplement: Supplementary file 1 [file ijms-26-04377-s001.zip › Figure S4A. InterProScan family distribution.png]

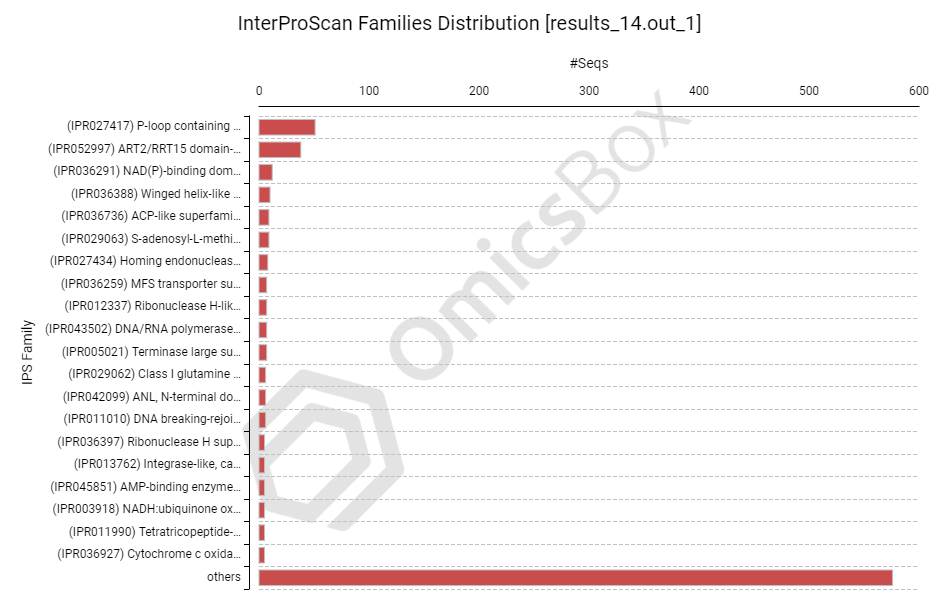

Supplement: Supplementary file 1 [file ijms-26-04377-s001.zip › Figure S4B. InterProScan family distribution.png]

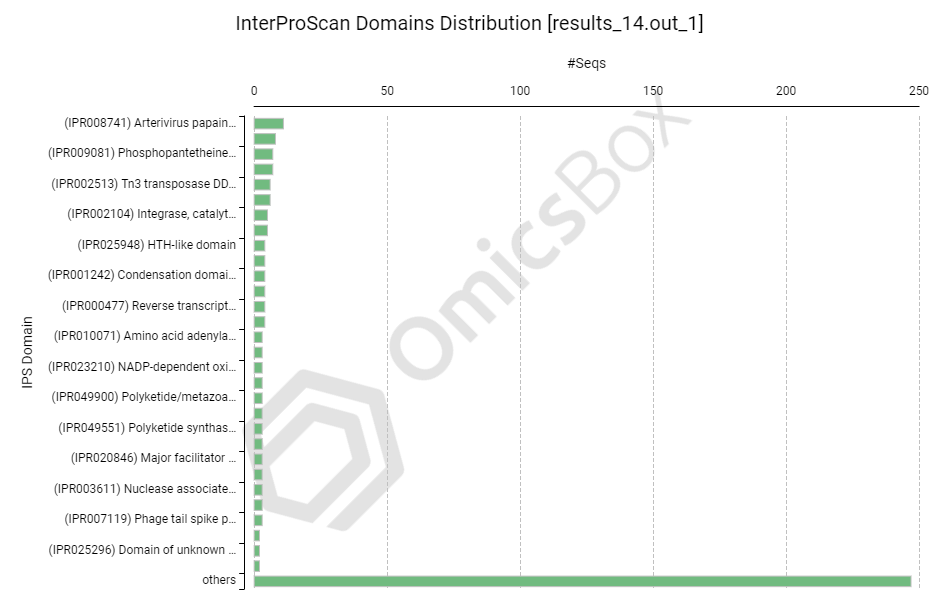

Supplement: Supplementary file 1 [file ijms-26-04377-s001.zip › Figure S5. InterProScan domains distribution.png]

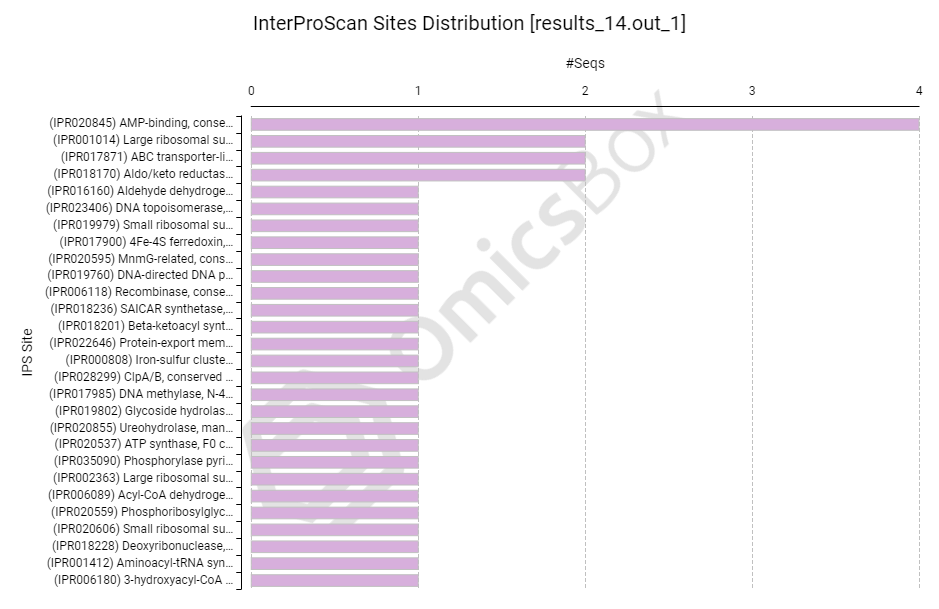

Supplement: Supplementary file 1 [file ijms-26-04377-s001.zip › Figure S6. InterProScan sites distribution.png]

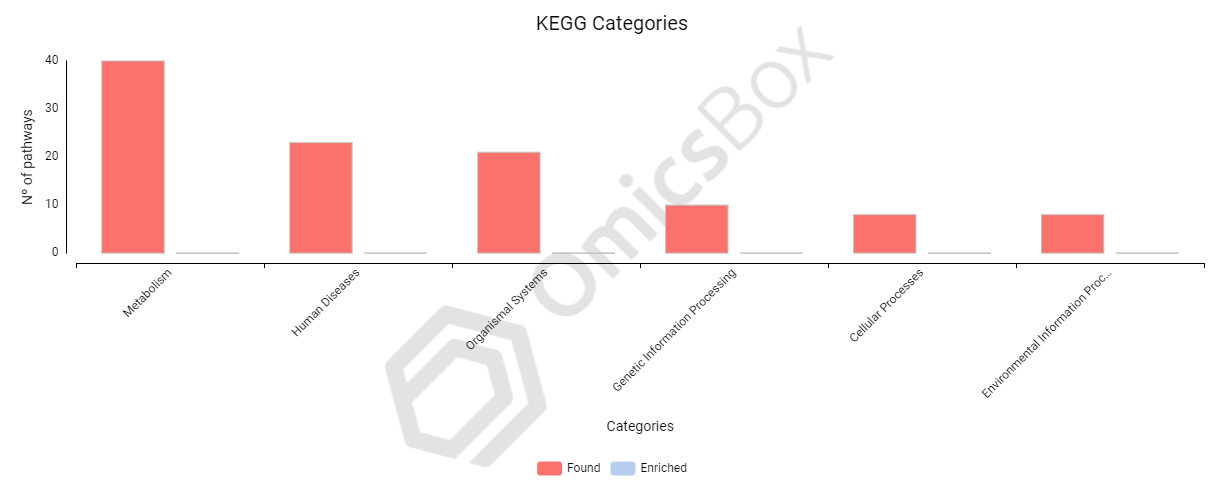

Supplement: Supplementary file 1 [file ijms-26-04377-s001.zip › Figure S7. KEGG categories pathways.png]

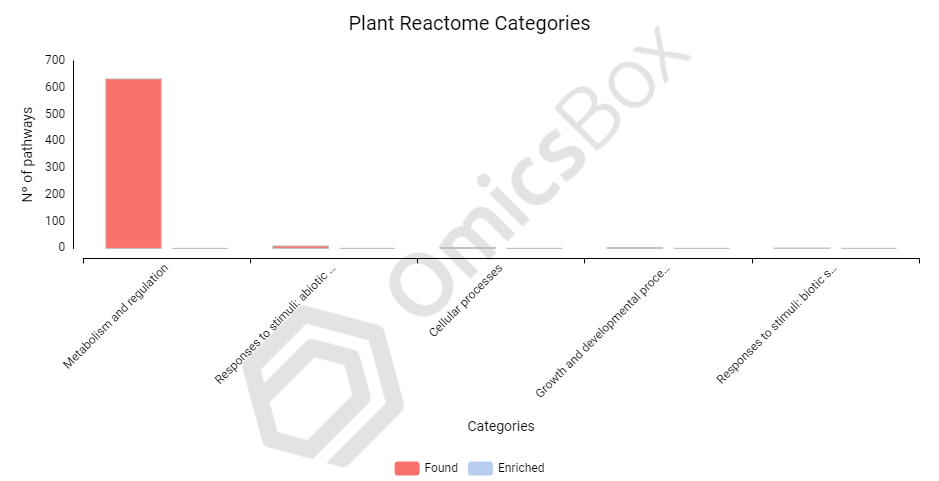

Supplement: Supplementary file 1 [file ijms-26-04377-s001.zip › Figure S8. Plant reactome pathways.png]

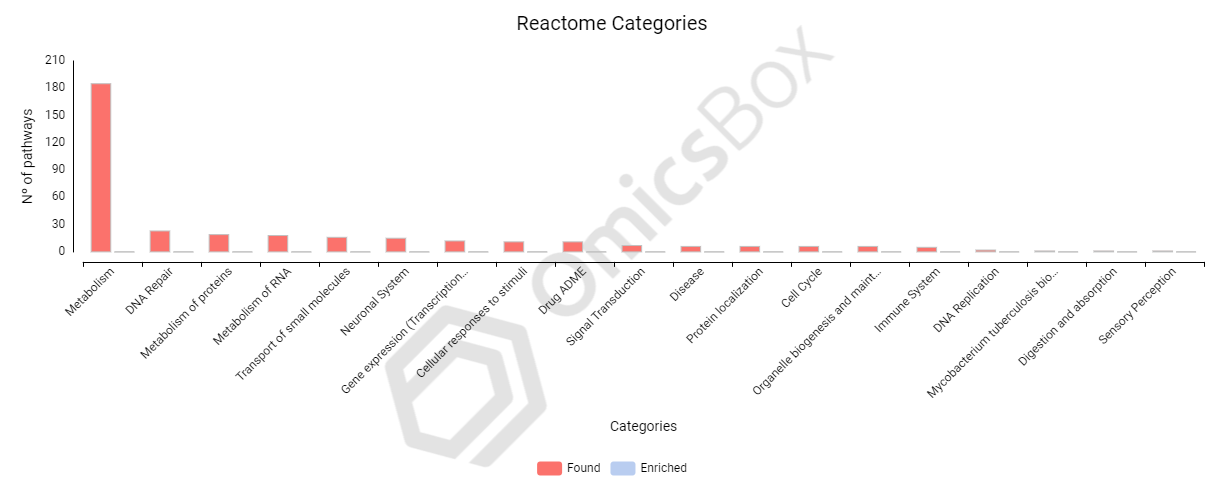

Supplement: Supplementary file 1 [file ijms-26-04377-s001.zip › Figure S9. Reactome pathways.png]
